# Supplementary material for: Genome-Wide Identification and Expression Analysis of the NLP Family in Sweet Potato and Its Two Diploid Relatives
Source: Int J Mol Sci. 2025 Aug 29;26(17):8435. doi: 10.3390/ijms26178435 (PMC12428392; doi:10.3390/ijms26178435)
Supplement: Supplementary file 1 [file ijms-26-08435-s001.zip › ijms-3840737-supplementary.pdf]

**Tabel S1.** Identification of *NLP* family genes in *I. batatas*, *I. trifida*, and *I. triloba*

| *Arabidopsis* | Homologous gene in *I.batatas/I. trifida/ I. triloba* | Gene ID | Gene name | Chromosome localization |
|---|---|---|---|---|
| Group I: *AT4G24020.1/AtNLP1* *AT1G20640.1/AtNLP2* *AT4G35270.1AtNLP3* *AT4G38340.1/AtNLP4* *AT2G43500.1AtNLP5* | *I. batatas* | *g38014* | *IbNLP6* | *LG9:30325182-30329672* |
| | | *g59844* | *IbNLP7* | *LG14:30719712-30725032* |
| | *I. trifida* | *itf09g00120.t1* | *ItfNLP3* | *Chr09:62967-67948* |
| | | *itf10g01180.t1* | *ItfNLP4* | *Chr10:971771-976115* |
| | | *itf15g10360.t1* | *ItfNLP8* | *Chr15:7261087-7267011* |
| | *I. triloba* | *itb09g00110.t1* | *ItbNLP7* | *Chr09:196047-201018* |
| | | *itb10g01040.t1* | *ItbNLP8* | *Chr10:757896-762050* |
| | | *itb15g10720.t1* | *ItbNLP12* | *Chr15:8423337-8428376* |
| Group II: *AT1G64530.1/AtNLP6* *AT3G59580.1/AtNLP7* | *I. batatas* | *g19466* | *IbNLP4* | *Chr05:20999118-21003180* |
| | *I. trifida* | *itf10g16290.t1* | *ItfNLP6* | *Chr10:18632056-18636241* |
| | | *itf12g17180.t1* | *ItfNLP7* | *Chr12:16821589-16825898* |
| | *I. triloba* | *itb10g16430.t1* | *ItbNLP10* | *Chr10:22676432-22680754* |
| | | *itb12g17910.t1* | *ItbNLP11* | *Chr12:220158134-20162369* |
| Group III: *AT1G76350.1/AtNLP8* *AT2G17150.1/AtNLP9* | *I. batatas* | *g36533* | *IbNLP5* | *Chr08:7215664-7220968* |
| | *I. trifida* | *itf15g15690.t1* | *ItfNLP9* | *Chr15:13493996-13499347* |
| | *I. triloba* | *itb10g09360.t1* | *ItbNLP9* | *Chr02:24313432-24315665* |
| Group IV | *I. batatas* | *g3955* | *IbNLP1* | *LG1:28560094-28561533* |
| | | *g4351* | *IbNLP2* | *LG2:1179338-1183553* |
| | | *g4375* | *IbNLP3* | *LG2:1344575-1347153* |
| | *I. trifida* | *itf04g33830.t1* | *ItfNLP1* | Chr04:31987284-31990152 |
| | | *itf04g33840.t1* | *ItfNLP2* | *Chr04:31991893-31995175* |
| | | *itf10g13430.t1* | *ItfNLP5* | *Chr10:16020142-16021431* |
| | *I. triloba* | *itb04g33470.t1* | *ItbNLP1* | Chr04:35709582-35714905 |
| | | *itb04g33480.t1* | *ItbNLP2* | *Chr04:35717076-35719440* |
| | | *itb04g33500.t1* | *ItbNLP3* | *Chr04:35738285-35740522* |
| | | *itb04g33510.t1* | *ItbNLP4* | *Chr04:35745513-35749754* |
| | | *itb04g33560.t1* | *ItbNLP5* | *Chr04:35764450-35767539* |
| | | *itb05g01640.t1* | *ItbNLP6* | *Chr05:1313452-1314975* |

**Table S2.** Primers used in this study.

| Gene | Forward Primer | Reverse Primer |
|---|---|---|
| *IbNLP1* | GGGGTTTCGATTCAATCCTAC | ATCCCGGGGTTCAAATCTG |
| *IbNLP2* | ATGGTTACATTTTCCAGCGAAC | GAGAGGCAGCATATGGTTCC |
| *IbNLP3* | ATGGCAATTCTTGAGAACCCC | CTATATTCTGTAACTGTTGAACCTCA |
| *IbNLP4* | ATGCCCGAACCGGATGA | GGCAGCGAAGATCTGATCG |
| *IbNLP5* | ATGTTAACGGCGTGGAATATTT | TTGAACACTTTGTACCATCCAATG |
| *IbNLP6* | AGAGGCAAAGAGCATCTCTTCTC | TGTAATCGAATTTCCAGTCTTTGG |
| *IbNLP7* | ATGGGAGATGGTTCCTCTGC | AAAAGTTGGATCCTTCAGTGGTC |
| *IbActin* | AGCAGCATGAAGATTAAGGTTGTAG | TGGAAAATTAGAAGCACTTCCTG |

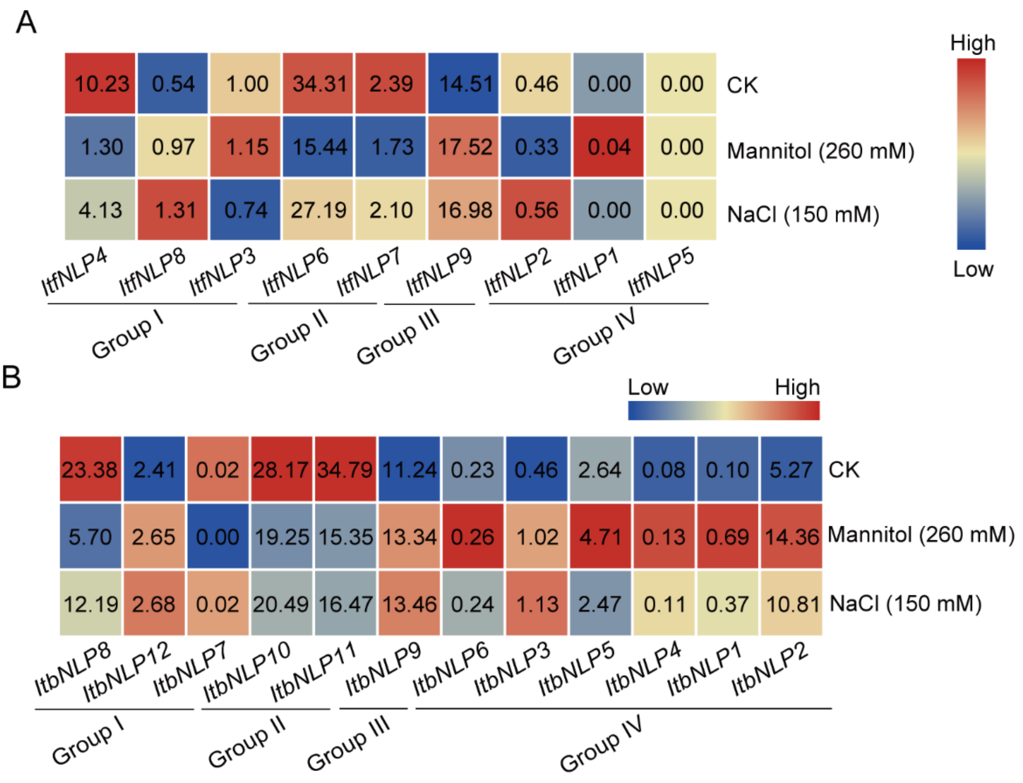


**Figure S1.** Gene expression patterns of *NLPs* in response to different abiotic stresses (Mannitol and NaCl) in *I. trifida* and *I. triloba* as determined by RNA-seq. $Log_2$ (FPKM) are shown in the boxes.
